# Baseline Predictors of the Long-Term Insufficient Biochemical Response in Patients with Autoimmune Hepatitis: A Single Center Experience

**DOI:** 10.3390/jcm12083008

**Published:** 2023-04-20

**Authors:** Pierluigi Toniutto, Michela Zorzi, Lorenzo D’Alì, Annarosa Cussigh, Sara Cmet, Davide Bitetto, Ezio Fornasiere, Elisa Fumolo, Carla Di Loreto, Edmondo Falleti

**Affiliations:** 1 Hepatology and Liver Transplantation Unit, Department of Specialized Medicine, Udine University Hospital, 33100 Udine, Italy; 2 Department of Medicine, Institute of Pathological Anatomy, Udine University Hospital, 33100 Udine, Italy; 3 Clinical Pathology, Udine University Hospital, 33100 Udine, Italy

**Keywords:** autoimmune hepatitis, azathioprine, complete biochemical response, liver cirrhosis

## Abstract

The treatment response criteria in autoimmune hepatitis (AIH) have been recently updated. This study aimed to assess treatment responses in 39 (16 males) patients with AIH confirmed by histology. Prednisone added to azathioprine or mycophenolate was the most frequent first-line treatment. Serum alanine aminotransferase (ALT) levels were periodically checked for a median of 45 months. Eight (20.5%) patients presented 4 weeks non-response (NR). Baseline lower multiples of ALT above the upper normal limit (UNL) (*p* = 0.005), Ishak liver fibrosis score > 3 (*p* = 0.029), and less frequent confluent necrosis > 2 (*p* < 0.001) were independent predictors of NR. 24 (61.5%) patients achieved complete biochemical response (CBR) at six months. Ishak liver fibrosis score ≤ 3 (*p* < 0.001), lobular eosinophilic infiltrate (*p* < 0.001), and ≥50% decrease in serum ALT levels at week 4 (*p* < 0.001) were independent predictors of CBR. In addition, the GLUCRE score, derived from the multiplication of serum creatinine (mg/dL) and glucose (mg/dL) levels, were identified. A baseline GLUCRE value > 100 strongly predicted CBR failure (*p* = 0.003) at a follow-up greater than 12 months. In conclusion, the absence of cirrhosis and a ≥50% UNL decrease in serum ALT levels were independent predictors for CBR. A baseline GLUCRE score may help identify patients maintaining longer CBR.

## 1. Introduction

Autoimmune hepatitis (AIH) is a relatively rare inflammatory disease of the liver caused by an aberrant self-immune response directed against liver tissue. The pathogenesis of AIH is related to environmental triggers, leading to loss of tolerance to self-liver antigens, particularly in genetically susceptible individuals [1,2]. In adults, the peak of AIH incidence is around 40–50 years, with the predominance of the female sex [3].

AIH is diagnosed by combining the observation of interface hepatitis on liver histology with increased serum transaminase and immunoglobulin G (IgG) concentrations and the presence of characteristic autoantibodies after the exclusion of viral, hereditary, metabolic, cholestatic, and drug-induced liver diseases that may resemble AIH [4]. The positivity of serum antinuclear antibodies (ANA) and/or smooth muscle antibodies (SMA)/anti-actin antibodies are characteristic of the adult form of the disease, named type 1 AIH, while the positivity of antibodies to liver-kidney microsomes (LKM) is characteristic of the pediatric form of the disease, called type 2 AIH [4,5,6].

Treatment of AIH is devoted to preventing disease progression and promoting the regression of fibrosis by the complete remission of histological disease activity and the normalization of serum IgG and transaminase levels [4,6]. The combination of corticosteroids with azathioprine (AZA) is the standard first-line treatment option recommended by the US and European clinical guidelines [4,6]. In cases that fail to achieve histological and/or biochemical response, the substitution of AZA with mycophenolate mofetil (MMF) and corticosteroids is recommended [4,6]. The definition of treatment response in AIH was first provided in 1993 by the International Autoimmune Hepatitis Group (IAIHG) [7] and subsequently revised in 2010 [8] and 2015 [6]. However, all treatment response definitions have been criticized because they are considered too complex and not uniform among clinical guidelines. More recently, IAIHG members performed a systematic review of response criteria and endpoints in AIH, aiming to provide a simple and reproducible framework to define treatment response and nonresponse irrespective of the therapeutic intervention [9] as follows: complete biochemical response (CBR) was defined as the normalization of serum transaminase and IgG levels below the upper normal limit (UNL) within six months after treatment initiation; insufficient response (IR) was defined as lack of CBR within six months after treatment initiation; and nonresponse (NR) was defined as a <50% decrease in serum transaminase levels within four weeks after treatment initiation. These new definitions of treatment responses should be used to set a global standard for reporting study results in AIH, enabling the comparison of results among clinical trials. Thus, the present study aimed to evaluate treatment response rates in a consecutive series of adult patients with type I AIH, adopting these updated criteria [9]. The primary endpoint of the present study was to assess the rates of NR, IR, and CBR. In addition, the maintenance of CBR was assessed throughout the entire patient follow-up. In addition, the secondary endpoint was to identify potential predictors of achieving and maintaining CBR in the long term.

## 2. Materials and Methods

### 2.1. Patients

All consecutive adult patients diagnosed with treatment-naïve type I AIH (*n* = 39) at the Hepatology and Liver Transplantation Unit of the University of Udine between 1 January 2009 and 31 December 2022 were enrolled in the present study. Diagnosis of type I AIH was made per the IAIHG criteria [5,10] in patients presenting increased serum transaminase and IgG levels associated with positive ANA and/or SMA or showing the characteristic features of interface hepatitis with plasma-cell infiltrations in liver biopsy. Genetic, viral, toxic, drug-related, and metabolic-related liver diseases were ruled out. All patients were evaluated every 3 to 6 months and followed for a median (IQR) of 45 (20–98) months by physical and laboratory examinations, including serum transaminase level measurements. All patients agreed to participate in the study, and the retrospective anonymous analyses of their clinical and demographic characteristics were approved by the Internal Review Board of the hospital in accordance with the 1975 Declaration of Helsinki.

### 2.2. Antibody Testing

ANA and SMA were measured by indirect immunofluorescence on fresh frozen sections of rodent multiorgan substrates. In addition, anti-liver cytosol type 1 and liver-kidney microsomal-1 (LKM-1) antibodies were measured by immunoblot or chemiluminescence methods.

### 2.3. Liver Histology

A baseline liver biopsy was performed in all patients. Liver specimens were formalin-fixed and reevaluated by an experienced liver immunopathologist (L.D.) using the recent histological criteria proposed by the IAIHG [11]. Only patients presenting the histologic criteria for likely AIH were selected for the analysis. The severity of AIH was graded using the modified hepatitis activity index (mHAI) [12].

### 2.4. Pharmacological Treatment Schedules

Patients received induction therapy for 30 days, either with 40 mg/day prednisone alone or in combination with 100 mg/day AZA, added two weeks after prednisone initiation. Afterwards, prednisone was tapered by 5 mg every two weeks until withdrawal. AZA-intolerant patients received 2 gr/day MMF, and prednisone-intolerant patients received 9 mg/day budesonide plus AZA. Drug intolerance was assessed by the treating physician and was defined as any drug-induced adverse event leading to potential drug discontinuation [9]. Treatment was discretionally modified by the treating physician based on achieving or not achieving CBR. The potential teratogenicity of MMF was explained to females of reproductive age, and all of them were counseled on strict and effective contraceptive measures during treatment.

### 2.5. Statistical Analysis

Statistical analysis was performed using Stata 15.1 statistical software (StataCorp. 2017. Stata Statistical Software: Release 15.1 College Station, TX, USA: StataCorp LLC.). Because normality testing of transaminases failed, a nonparametric rank-sum (Mann-Whiney) test was used. Data are presented as medians and interquartile (IQR) ranges. Categorical variables were compared using the Pearson chi-square test, and data were presented as frequencies (%). Forward-stepping logistic regression analysis was used to select independent predictors for the 4-week nonresponse, 6-month, and last follow-up complete biochemical response. All variables showing a *p*-value ≤ 0.10 in the univariate analysis were included. Pseudo R^2^, the area under the ROC curve, and the percentage of correct classification are presented as quality estimations of the regression model. Interaction (additive or multiplicative) effects between variables were explored following Stata documentation. A cutoff value equal to 100 for the new parameter constructed from the multiplication of serum creatinine (mg/dL) and glucose (mg/dL) levels (GLUCRE) was obtained with the aid of ROC curve analysis.

## 3. Results

In total, 39 adult patients were enrolled in the present study. The main demographic and clinical characteristics of the studied population are shown in Table 1. The median age of the patients was 55 years, and 59% of the patients were females. The median serum transaminase levels at presentation displayed a large range because 51% of patients presented an acute form of AIH (aminotransferases > 10× UNL). Regarding the histologic characteristics of AIH at presentation, liver fibrosis was absent or mild in most patients because severe liver fibrosis or cirrhosis was present in only 7 (18%) patients (Table 2).

### Pharmacological Treatment and Patterns of Response

Induction therapy with prednisone alone or added to AZA was adopted in 18 (46.2%) and 16 (41%) patients, respectively. However, three (7.7%) patients were intolerant to AZA immediately after the first administration; therefore, MMF was used in combination with prednisone in these patients, and two (5.2%) patients, diabetes and osteoporosis prompted the treating physician to prescribe budesonide, instead of prednisone, in addition to AZA.

Eight (20.5%) patients presented NR. In the multivariate analysis, baseline lower multiples of serum alanine aminotransferase (ALT) levels above the UNL (*p* = 0.005), Ishak fibrosis score > 3 at liver biopsy (*p* = 0.029), and less frequent confluent necrosis > 2 (*p* < 0.001) were selected as independent predictors of NR (Table 3). Interestingly, the type of treatment was not associated with NR in either the univariate or multivariate analysis.

**Figure 1:**
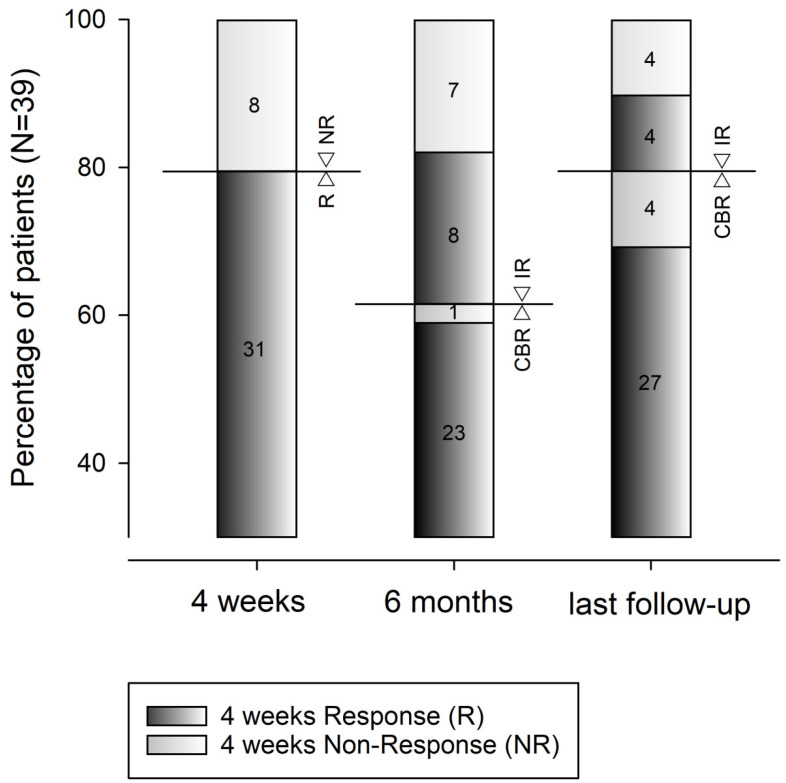
Evolving patterns of responses to immunosuppressive treatment in patients with autoimmune hepatitis (*n* = 39). Horizontal bars and arrowheads delimit the percentages of response. The dark gray histograms (with the number of patients reported inside) show the percentage of patients obtaining the response (R) to treatment, defined as a ≥50% decrease from baseline of serum alanine aminotransferase and aspartate aminotransferase levels at four weeks. The light gray histograms (with the number of patients reported inside) show the percentage of patients presenting nonresponse (NR) to treatment, defined as a <50% decrease from baseline of serum alanine aminotransferase and aspartate aminotransferase levels. Complete biochemical response (CBR) and insufficient response (IR) were evaluated both at six months and at the last (IQR) follow-up time of 45 (20–98) months.

CBR at six months was achieved by 24 (61.5%) patients. Among the 31 patients presenting a ≥50% decrease in serum transaminase levels at four weeks, which were defined as responders (R), 23 (74.2%) patients achieved CBR at six months. In contrast, only 1 of 8 (12.5%) NR patients achieved CBR at six months (Figure 1). Achieving a ≥50% decrease in serum ALT levels at week 4 (*p* < 0.001), Ishak fibrosis score ≤ 3 (*p* < 0.001), and lobular eosinophilic infiltrate (*p* < 0.001) were selected as independent predictors of achieving CBR at six months. Similar to what was observed in the analysis evaluating factors associated with IR, prednisone maintenance was not predictive of achieving CBR at six months (Table 4). Therefore, in the 15 (38.5%) patients who presented with IR at six months, the following changes were made to the treatment regimens: prednisone was reintroduced in addition to AZA or MMF in 13 patients, and AZA was replaced by MMF monotherapy in 2 patients.

**Figure 2:**
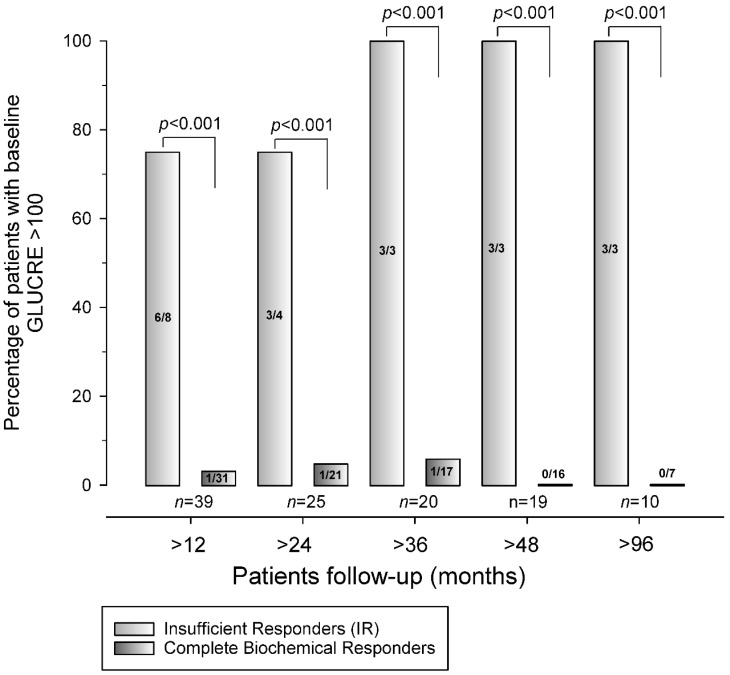
Correlation between the GLUCRE score [obtained by the multiplication of serum creatinine (mg/dL) and glucose (mg/dL) levels] > 100 and the achievement of complete biochemical response (CBR) at different times of follow-up.

At the end of the follow-up, 31 (79.5%) patients fulfilled the criteria for CBR (Figure 1). At baseline, a higher multiple of ALT values of the UNL was the only biochemical predictor for achieving CBR. To identify a combination of baseline demographic, histologic, or biochemical parameters predicting the achievement of CBR at the end of follow-up, a new parameter derived from the multiplication of serum creatinine (mg/dL) and glucose (mg/dL) levels (GLUCRE) was constructed. A GLUCRE value > 100 was present in 1 of 31 (3.2%) patients who achieved CBR at the end of follow-up compared to 6 of 8 (75%) patients who did not achieve CBR at the end of follow-up (*p* < 0.001). In the multivariate analysis, GLUCRE > 100 was identified as a stronger predictor of CBR failure (*p* = 0.003) at the end of the follow-up (Table 5). Figure 2 illustrates the association between a baseline GLUCRE value > 100 and CBR failure. For any interval starting from more than 12 months and up to 96 months of follow-up, the baseline GLUCRE > 100 was always significantly associated with CBR failure (*p* < 0.001 for all time frames).

## 4. Discussion

In the present study, the female/male ratio of patients presenting type 1 AIH was 2.4, and the median age was 55. Furthermore, 51.3% of patients had an acute presentation of the disease. These data agreed with the recent data reported in Europe and other countries [6,13,14]. All patients underwent liver biopsy for AIH diagnosis confirmation according to guidelines [4], which allowed the exclusion of patients with overlapping syndromes but the inclusion of five patients who were ANA negative at presentation. ANA or SMA was absent in 19–34% of patients originally diagnosed with cryptogenic hepatitis and then reclassified as AIH by liver histology [15,16].

NR to treatment was recorded in 8 of the 39 (20.5%) patients, which agreed with a recent report including a large cohort of patients from Greece [17], in which the NR was 19.3% in patients treated with AZA and 7.7% in those treated with MMF. The present study showed no significant differences in NR rates between patients treated with AZA and those treated with MMF. This may be due to MMF being administered as induction therapy in only three patients. In the multivariate analysis, the independent predictors of NR were Ishak fibrosis score > 3, confluent necrosis score ≤ 2 at liver biopsy, and lower multiple of UNL of serum ALT levels. The importance of liver fibrosis severity in increasing the NR probability has been confirmed in a large cohort of Chinese patients with AIH [18]. NR was more frequently observed in patients with cirrhosis at presentation.

In the present study, CBR was observed in 61.5% of patients, which agreed with the findings of a recent cohort of Portuguese patients [19]. However, in Portuguese patients, advanced liver fibrosis or cirrhosis at baseline was not selected as a predictor for achieving CBR. This difference may be explained by the higher prevalence of advanced liver fibrosis/cirrhosis (nearly 50%) in the Portuguese patients compared to that observed in the present study (18%). As a result, the rate of CBR obtained in the present patients was lower than that reported in Chinese [18] and Greek [17] patients with AIH, who developed CBR in 77.2% and 78.9% of cases, respectively. Similarly, the percentage of patients presenting a ≥50% decrease in serum ALT levels at four weeks who achieved CBR at six months was higher in the previous Chinese population (79%) than in the present patients (74.2%). These differences may be due to the higher percentage of male patients and the low number of patients receiving MMF in the present study, which has been associated with better CBR rates than patients receiving AZA [17]. The absence of severe liver fibrosis and a ≥50% decrease in serum ALT levels at week four were selected as independent predictors for achieving CBR at six months, which agreed with the results in a Chinese population [18]. CBR increased to 79.5% in the present study at the end of follow-up, which agreed with the findings in Portuguese patients after a median follow-up time of 6.5 years [19]. This result may be attributable to the change in therapy that occurred in the 15 patients who presented an IR; seven of these patients achieved CBR, and all received MMF as a second-line treatment. This observation confirmed recent data showing that first- or second-line treatment with MMF compared to AZA increases the probability of achieving CBR at 12 months [17,20].

Despite a large number of previously published AIH studies, no clear baseline predictors of long-term CBR have been demonstrated thus far. Older age, HLA DRB1*04:01 mutant, and higher serum ferritin levels [21] have been advocated as predictors of biochemical response [4,5,6,7,8] but with conflicting results [22]. The present study developed a novel baseline GLUCRE score, which selected most patients failing to achieve CBR evaluated after 12 months and during the entire follow-up period. No data are available thus far regarding the additional effect of serum glucose and creatinine levels in influencing treatment response in AIH. In a mice model of Concanavalin-A-induced hepatitis, which mimics AIH, regulatory T-cells (Tregs) in the liver showed an impaired immunosuppressive function, as reflected by the downregulation of mRNA levels of several anti-inflammatory cytokines [23]. Interestingly, the functional impairment of Tregs has been attributed to enhanced glycolysis and glucose metabolism reprogramming. This observation suggests that impaired glucose metabolism could abolish the immunosuppressive effect exerted by normal functioning Tregs, leading to the maintenance of hepatic inflammation resistant to immunosuppressive treatment in AIH [23]. A higher model of end-stage liver disease (MELD) score has been recently identified as an early independent predictor of corticosteroid response in clinically severe forms of AIH [24]. In agreement with the present study, the previous study reported that the baseline serum creatinine levels, evaluated outside of the MELD score, are significantly higher in non-responders than in responders. Although no differences in response rates were observed between patients with and without diabetes, the serum glucose levels were not evaluated [24]. In the present study, a GLUCRE score > 100 in selected patients presenting an additional detrimental effect of serum creatinine and glucose levels may have been attributed to the low probability of achieving CBR.

The present study had some limitations. The number of patients was small. A second liver biopsy or periodical transient elastography (TE) assessment was not performed to evaluate the response to treatment and/or liver fibrosis regression. However, a liver biopsy performed two years after treatment initiation is not mandatory and has been suggested to select patients in whom drug withdrawal is considered [4]. Although CBR was strongly linked to regression of liver stiffness [25], there are no clear indications on the use of TE to decide which patient’s immunosuppressive therapy can be discontinued. Biochemical recurrence has been demonstrated in up to 50% of patients after treatment discontinuation, irrespective of previous CBR and/or histologic remission of the disease [26]. Thus, we intended to indefinitely maintain the minimum required immunosuppressive treatment as suggested by several experts [27].

In summary, the findings confirmed that the three new AIH treatment response criteria proposed by Pape et al. [9] are reproducible and useful to consistently compare the results obtained in treating AIH between different studies. Furthermore, the data confirmed that the absence of cirrhosis at baseline and ≥50% of UNL decrease in serum ALT levels at week four are independent predictors for achieving CBR. If confirmed in multi-centre studies, enrolling a larger number of patients, the baseline GLUCRE score may help identify patients who will maintain a longer CBR.

## Figures and Tables

**Table 1:** Baseline demographic and biochemical characteristics of the studied population. Categorical variables are reported as frequencies (%), and continuous variables are reported as medians (interquartile range).

	Patients (*n* = 39)
Age (years)	55.0 (46.4–64.4)
Female gender	23 (59.0)
MELD score	10.1 (7.8–15.3)
Leukocytes (*n*·1000/μL)	8.05 (5.85–11.4)
Hemoglobin (g/dL)	13.6 (12.5–14.7)
Platelets (*n*·1000/μL)	208 (174–257)
Glucose (mg/dL)	86 (80–96)
eGFR (ml/min/1.73 m^2^)	81 (69–88)
Aspartate aminotransferase (IU/mL)	235 (91–590)
Alanine aminotransferase (IU/mL)	333 (101–843)
γ-Glutamyl-transpeptidase (IU/mL)	166 (81–275)
Alkaline Phosphatase (IU/mL)	127 (86–173)
Total bilirubin (mg/dL)	1.62 (0.80–6.66)
Conjugated bilirubin (mg/dL)	0.97 (0.38–4.26)
Albumin (g/dL)	3.69 (3.38–4.14)
γ-Globulins (g/dL)	1.76 (1.39–2.23)
INR	1.11 (1.03–1.22)
ANA positivity	36 (92.3)
ASMA positivity	39 (100)
Use of medications	29 (74.4%)
Concurrent autoimmune diseases	
Autoimmune thyroiditis	2 (5.1)
Other	4 (10.3)

MELD: a model of end-stage liver disease; eGFR: estimated glomerular filtration rate; INR: international normalized ratio; ANA: antinuclear antibody; ASMA: anti-smooth muscle antibody.

**Table 2:** Histologic characteristics of liver biopsies performed in patients at disease presentation. Categorical variables are reported as frequencies (%), and continuous variables are reported as medians (interquartile range).

	Patients (*n* = 39)
Portal tracts (number)	13 (9–16)
Ishak fibrosis score	
0–4	32 (82)
5–6	7 (18)
Perivenular and/or centrilobular fibrosis	8 (20.5%)
Piecemeal necrosis	
0–2	23 (59)
3–4	16 (41)
Confluent necrosis	
0–2	29 (74.3)
3–4	10 (25.7)
Focal lytic necrosis	
0–2	19 (48.7)
3–4	20 (51.3)
Portal inflammation	
0–2	24 (61.5)
3–4	15 (38.5)
Portal lymphocytic/lympho-plasmocytic infiltrate	36 (92.3)
Portal eosinophilic infiltrate	34 (87.2)
Lobular lymphocytic/lympho-plasmocytic infiltrate	28 (71.8)
Lobular eosinophilic infiltrate	13 (33.3)
Ductular reaction	15 (38.5)
Portal venous/centrilobular endotheliitis	31 (79.5)
Hepatic rosettes	31 (79.5)
Emperipolesis	23 (59.0)
Cholestasis	4 (10.3)
Cholangitis	1 (2.6)

**Table 3:** Association among baseline demographic, clinical, and histological characteristics of patients (*n* = 39) concerning the type of response to treatment evaluated at four weeks. Response and no response (NR) to treatment were defined as having or not having a ≥50% decrease from baseline in serum alanine aminotransferase and aspartate aminotransferase levels, respectively. The categorical parameters are presented as frequencies (%), and the Pearson chi-squared test was used for statistical comparisons. Continuous variables are presented as medians (interquartile range), and the rank-sum test (Mann-Whitney) was used for statistical comparisons. Stepwise regression with a forward approach was used to discriminate independent predictive variables associated with nonresponse.

	Univariate Analysis			Multivariate Analysis		
	NR	Response				
	*n* = 8 (20.5)	*n* = 31 (79.5)	*p*	OR	95% CI	*p*
Male gender	3 (37.5)	13 (41.9)	0.820			
Age (years at diagnosis)	50 (39–64)	56 (47–65)	0.509			
Body mass index (kg/m^2^)	27 (25–37.5)	25.5 (24–29)	0.228			
MELD score	8.6 (7.8–10.2)	11.0 (7.8–15.3)	0.741			
Leukocytes (*n*·1000/μL)	10.7 (5.55–12.7)	7.71 (5.85–10.6)	0.313			
Platelets (*n*·1000/μL)	175 (111–237)	212 (176–268)	0.149			
Aspartate aminotransferase (UNL)	2.67 (2.26–5.94)	7.50 (3.50–15.9)	0.035	-	-	-
Alanine aminotransferase (UNL)	2.49 (1.18–7.69)	17.2 (7.00–25.5)	0.002	1.141	1.039–1.251	0.005
γ-Glutamyl-transpeptidase (UNL)	3.45 (2.33–5.77)	3.26 (1.77–6.00)	0.676			
Alkaline Phosphatase (UNL)	0.97 (0.83–1.71)	1.03 (0.82–1.56)	0.932			
Glucose (mg/dL)	96 (84–107)	86 (78–94)	0.174			
eGFR (ml/min/1.73 m^2^)	74 (57–90)	82 (71–87)	0.531			
Total bilirubin (mg/dL)	1.21 (0.55–4.92)	1.78 (0.80–6.66)	0.313			
Conjugated bilirubin (mg/dL)	0.45 (0.32–2.39)	1.37 (0.38–4.27)	0.239			
Albumin (g/dL)	3.07 (2.90–3.75)	3.76 (3.42–4.30)	0.038	-	-	-
γ-Globulins (g/dL)	2.18 (1.40–2.65)	1.76 (1.39–2.17)	0.414			
INR	1.13 (1.03–1.30)	1.10 (1.03–1.21)	0.715			
ANA titer (>1:640)	2 (25.0)	3 (9.7)	0.248			
ASMA titer (>1:80)	3 (37.5)	7 (22.6)	0.389			
Ishak fibrosis score > 3	5 (62.5)	2 (6.5)	<0.001	0.102	0.013–0.789	0.029
Perivenular and/or centrilobular fibrosis	1 (12.5)	7 (22.6)	0.529			
Interface hepatitis score > 2	4 (50.0)	12 (38.7)	0.563			
Confluent necrosis score > 2	0 (0.0)	10 (32.3)	0.062	>100	>100–>100	<0.001
Focal lytic necrosis score > 2	4 (50.0)	16 (51.6)	0.935			
Portal inflammation score > 2	4 (50.0)	11 (35.5)	0.452			
Portal lymphocytic/lymphoplasmacytic infiltrate	6 (75.0)	30 (96.8)	0.039	-	-	-
Portal eosinophilic infiltrate	6 (75.0)	28 (90.3)	0.248			
Lobular lymphocytic/lymphoplasmacytic infiltrate	6 (75.0)	22 (71.0)	0.821			
Lobular eosinophilic infiltrate	2 (25.0)	11 (35.5)	0.575			
Ductular reaction	2 (25.0)	13 (441.9)	0.380			
Portal venous/centrilobular endotheliitis	5 (62.5)	26 (83.9)	0.182			
Hepatic rosettes	5 (62.5)	26 (83.9)	0.182			
Emperipolesis	5 (62.5)	18 (58.1)	0.820			
Cholestasis	2 (25.0)	2 (6.5)	0.123			
Induction treatment schedules						
Prednisone or budesonide	5 (62.5)	14 (45.2)	0.382			
Prednisone or budesonide + AZA	2 (25.0)	15 (48.4)	0.234			
Prednisone + MMF	1 (12.5)	2 (6.5)	0.567			

MELD: a model of end-stage liver disease; eGFR: estimated glomerular filtration rate; INR: international normalized ratio; ANA: antinuclear antibody; ASMA: anti-smooth muscle antibody; AZA: azathioprine; MMF: mycophenolate mofetil. Logistic model estimation parameters: pseudo R^2^ = 0.436; area under the ROC curve = 0.887; correct classification = 89.7%.

**Table 4:** Association between baseline demographic, clinical, and histological characteristics of patients (*n* = 39) concerning the achievement of complete biochemical response (CBR) or insufficient response (IR), defined as the normalization of serum transaminases and IgG levels below the upper normal limit (UNL) within six months after treatment initiation and as lack of CBR, respectively. Categorical parameters are presented as frequencies (%), and the Pearson chi-squared test was used for statistical comparisons. Continuous variables are presented as medians (interquartile range), and the rank-sum test (Mann-Whitney) was used for statistical comparisons. Stepwise regression with a forward approach was used to discriminate independent predictive variables associated with the achievement of CBR.

	Univariate Analysis			Multivariate Analysis		
	IR	CBR				
	*n* = 15 (38.5)	*n* = 24 (61.5)	*p*	OR	95% CI	*p*
Age (years at diagnosis)	52 (44–61)	58 (47–65)	0.341			
Male gender	4 (25.7)	12 (50.0)	0.150			
Body mass index (kg/m^2^)	26 (25–31)	25 (24–28.5)	0.361			
MELD score	10.2 (8.4–13.8)	8.7 (7.5–16.0)	0.479			
Leukocytes (*n*·1000/μL)	7.71 (5.10–11.8)	8.07 (5.95–10.9)	0.977			
Platelets (*n*·1000/μL)	214 (139–257)	205 (178–255)	0.438			
Aspartate aminotransferase (UNL)	6.13 (2.0–24.1)	5.89 (3.13–13.8)	0.697			
Alanine aminotransferase (UNL)	9.58 (2.45–24.7)	10.5 (4.77–22.95)	0.525			
γ-Glutamyl-transpeptidase (UNL)	2.51 (1.77–6.41)	3.31 (1.94–5.61)	0.806			
Alkaline Phosphatase (UNL)	1.33 (0.83–1.66)	1.00 (0.79–1.50)	0.488			
Glucose (mg/dL)	94 (85–110)	86 (78–89)	0.088	-	-	-
eGFR (ml/min/1.73 m^2^)	75 (59–88)	83 (72–88)	0.453			
Total bilirubin (mg/dL)	1.62 (0.97–3.91)	1.62 (0.75–7.00)	0.817			
Conjugated bilirubin (mg/dL)	0.97 (0.42–3.1)	1.15 (0.35–4.61)	0.966			
Albumin (g/dL)	3.67 (2.92–3.80)	3.72 (3.44–4.32)	0.089	-	-	-
γ-Globulins (g/dL)	2.17 (1.52–2.56)	1.71 (1.32–2.07)	0.076	-	-	-
INR	1.11 (1.05–1.26)	1.11 (1.03–1.20)	0.488			
ANA titers (>1:640)	2 (13.3)	3 (12.5)	0.940			
ASMA titers (>1:80)	5 (33.3)	5 (20.8)	0.384			
Ishak fibrosis score > 3	6 (40.0)	1 (4.2)	0.005	<0.01	<0.01–<0.01	<0.001
Perivenular and/or centrilobular fibrosis	2 (13.3)	6 (25.0)	0.308			
Interface hepatitis score > 2	5 (33.3)	11 (45.8)	0.440			
Confluent necrosis score > 2	3 (20.0)	7 (29.2)	0.524			
Focal lytic necrosis score > 2	6 (40.0)	14 (58.3)	0.265			
Portal inflammation score > 2	5 (33.3)	10 (41.7)	0.603			
Portal lymphocytic/lymphoplasmacytic infiltrate	12 (80.0)	24 (100)	0.023	-	-	-
Portal eosinophilic infiltrate	12 (80.0)	22 (91.7)	0.289			
Lobular lymphocytic/lymphoplasmacytic infiltrate	10 (66.7)	18 (75.0)	0.574			
Lobular eosinophilic infiltrate	1 (6.7)	12 (50.0)	0.005	>100	>100–>100	<0.001
Ductular reaction	6 (40.0)	9 (37.5)	0.876			
Portal venous/centrilobular endotheliitis	10 (66.7)	21 (87.5)	0.117			
Hepatic rosettes	10 (66.7)	21 (87.5)	0.117			
Emperipolesis	7 (46.7)	16 (66.7)	0.217			
Cholestasis	2 (13.3)	2 (8.3)	0.617			
Maintenance of steroid treatment	10 (66.7)	10 (41.7)	0.129			
Decreased ≥50% serum ALT levels at week 4	8 (53.3)	23 (95.8)	0.001	>100	>100–>100	<0.001

MELD: a model of end-stage liver disease; eGFR: estimated glomerular filtration rate; INR: international normalized ratio; ANA: antinuclear antibody; ASMA: anti-smooth muscle antibody. Logistic model estimation parameters: pseudo R^2^ = 0.525; area under the ROC curve = 0.889; correct classification = 82.1%.

**Table 5:** Association among baseline demographic, clinical, and histological characteristics of patients (*n* = 39) concerning the achievement of >12 months (CBR) or insufficient response (IR) defined as the normalization of serum transaminases and IgG levels below the upper normal limit (UNL) and as lack of CBR, respectively. Categorical parameters are presented as frequencies (%), and the Pearson chi-squared test was used for statistical comparisons. Continuous variables are presented as medians (interquartile range), and the rank-sum test (Mann-Whitney) was used for statistical comparisons. Stepwise regression with a forward approach was used to discriminate independent predictive variables associated with the achievement of CBR.

	Univariate Analysis			Multivariate Analysis		
	IR	CBR				
	*n* = 8 (20.5)	*n* = 31 (79.5)	*p*	OR	95% CI	*p*
Age (years at diagnosis)	55 (43–68)	55 (46–64)	0.807			
Male gender	4 (50)	12 (38.7)	0.563			
Body mass index (kg/m^2^)	25 (24–28)	26 (24–29)	0.625			
MELD score	8.5 (7.7–10.5)	11.1 (7.8–16.8)	0.414			
Leukocytes (*n*·1000/μL)	7.90 (6.47–12.7)	8.05 (5.62–11.2)	0.550			
Platelets (*n*·1000/μL)	190 (121–266)	208 (176–241)	0.404			
Aspartate aminotransferase (UNL)	3.25 (1.50–7.02)	7.31 (3.41–15.0)	0.106			
Alanine aminotransferase (UNL)	4.90 (1.82–9.86)	13.1 (5.21–25.6)	0.035	1.064	1.001–1.132	0.047
γ-Glutamyl-transpeptidase (UNL)	3.65 (1.77–6.55)	3.26 (1.80–5.23)	0.768			
Alkaline Phosphatase (UNL)	1.63 (0.75–1.87)	1.01 (0.82–1.55)	0.384			
Glucose (mg/dL)	104 (95–121)	86 (77–87)	<0.001	-	-	-
eGFR (mL/min/1.73 m^2^)	64 (53–80)	83 (71–88)	0.060	-	-	-
Total bilirubin (mg/dL)	0.89 (0.64–1.70)	2.59 (0.84–7.33)	0.154			
Conjugated bilirubin (mg/dL)	0.44 (0.31–0.82)	1.94 (0.38–4.27)	0.135			
Albumin (g/dL)	3.82 (3.19–4.40)	3.67 (3.38–4.10)	0.715			
γ-Globulins (g/dL)	1.94 (1.30–2.39)	1.76 (1.44–2.21)	0.917			
INR	1.08 (1.03–1.17)	1.11 (1.03–1.22	0.531			
ANA titers (>1:640)	3 (37.5)	2 (6.45)	0.019	-	-	-
ASMA titers (>1:80)	2 (25)	8 (25.8)	0.963			
Ishak fibrosis score > 3	3 (37.5)	4 (12.9)	0.106			
Perivenular and/or centrilobular fibrosis	0 (0.0)	8 (25.8)	0.107			
Interface hepatitis score > 2	5 (62.5)	11 (35.5)	0.166			
Confluent necrosis score > 2	2 (25)	8 (25.8)	0.963			
Focal lytic necrosis score > 2	3 (37.5)	17 (54.8)	0.382			
Portal inflammation score > 2	5 (62.5)	10 (32.3)	0.117			
Portal lymphocytic/lymphoplasmacytic infiltrate	7 (87.5)	29 (93.6)	0.567			
Portal eosinophilic infiltrate	6 (75.0)	28 (90.3)	0.248			
Lobular lymphocytic/lymphoplasmacytic infiltrate	6 (75.0)	22 (71.0)	0.821			
Lobular eosinophilic infiltrate	2 (25.0)	11 (35.5)	0.575			
Ductular reaction	3 (37.5)	12 (38.7)	0.950			
Portal venous/centrilobular endotheliitis	7 (87.5)	24 (77.4)	0.529			
Hepatic rosettes	7 (87.5)	24 (77.4)	0.529			
Emperipolesis	5 (62.5)	18 (58.1)	0.820			
Cholestasis	1 (12.5)	3 (9.7)	0.815			
Maintenance of steroid treatment	6 (75.0)	14 (45.2)	0.132			
GLU-CRE > 100	6 (75.0)	1 (3.2)	<0.001	0.015	0.001–0.233	0.003
Decreased ≥50% serum ALT levels at week 4	4 (50.0)	27 (87.1)	0.021	-	-	-
CBR at six months	2 (25.0)	22 (71.0)	0.017	-	-	-

MELD: a model of end-stage liver disease; eGFR: estimated glomerular filtration rate; INR: international normalized ratio; ANA: antinuclear antibody; ASMA: anti-smooth muscle antibody; GLUCRE: glucose (mg/dL) × creatinine (mg/dL). Logistic model estimation parameters: pseudo R^2^ = 0.501; area under the ROC curve = 0.914; correct classification = 92.3%.

## Data Availability

Data are available on request.
